# The Modulation of Gut Microbiota Composition in the Pathophysiology of Gestational Diabetes Mellitus: A Systematic Review

**DOI:** 10.3390/biology10101027

**Published:** 2021-10-11

**Authors:** Thubasni Kunasegaran, Vinod R. M. T. Balasubramaniam, Valliammai Jayanthi T. Arasoo, Uma Devi Palanisamy, Amutha Ramadas

**Affiliations:** Jeffrey Cheah School of Medicine and Health Sciences, Monash University Malaysia, Bandar Sunway 47500, Malaysia; thubasni.kunasegaran@monash.edu (T.K.); vinod.balasubramaniam@monash.edu (V.R.M.T.B.); t.jayanthi@monash.edu (V.J.T.A.); umadevi.palanisamy@monash.edu (U.D.P.)

**Keywords:** microbiome, 16S rRNA, metagenomics, gestational diabetes mellitus

## Abstract

**Simple Summary:**

Recent studies have placed a great deal of emphasis on the importance of the microbiome, especially the link between the alteration of gut microbiota and multiple associated diseases. Gut microbiota changes in pregnancy have a significant impact on metabolic function and may contribute to gestational diabetes mellitus (GDM). Although GDM carries long-term health risks that affect women, there are also significant short- and severe long-term consequences for the offspring. Regardless, there is a notable lack of research focusing on the impact of prominent microorganisms involved in the development of GDM. A comprehensive review was conducted to gather relevant data on the types of microorganisms that have been associated with GDM. The review found that certain microorganisms impact the onset and progression of GDM during pregnancy. Several bacterial strains associated with GDM are influenced by a diet high in fat and low in fiber. Therefore, integrating the idea of a microbiome-based individualized dietary intervention into gestational diabetes management may be incredibly beneficial.

**Abstract:**

General gut microbial dysbiosis in diabetes mellitus, including gestational diabetes mellitus (GDM), has been reported in a large body of literature. However, evidence investigating the association between specific taxonomic classes and GDM is lacking. Thus, we performed a systematic review of peer-reviewed observational studies and trials conducted among women with GDM within the last ten years using standard methodology. The National Institutes of Health (NIH) quality assessment tools were used to assess the quality of the included studies. Fourteen studies investigating microbial interactions with GDM were found to be relevant and included in this review. The synthesis of literature findings demonstrates that Bacteroidetes, Proteobacteria, Firmicutes, and Actinobacteria phyla, such as *Desulfovibrio*, Ruminococcaceae, *P. distasonis,* Enterobacteriaceae, *Collinsella*, and *Prevotella,* were positively associated with GDM. In contrast, *Bifidobacterium* and *Faecalibacterium*, which produce butyrate, are negatively associated with GDM. These bacteria were associated with inflammation, adiposity, and glucose intolerance in women with GDM. Lack of good diet management demonstrated the alteration of gut microbiota and its impact on GDM glucose homeostasis. The majority of the studies were of good quality. Therefore, there is great potential to incorporate personalized medicine targeting microbiome modulation through dietary intervention in the management of GDM.

## 1. Introduction

The microbiome is related to the pathogenesis of most chronic illnesses. Recent research has shown that gut microbiota plays a vital role in diabetes pathogenesis [1,2,3], cardiovascular disease [4], and obesity [5], as well as in therapeutic targets [6]. This also applies to gestational diabetes mellitus (GDM).

GDM affects roughly 25.1% of pregnancies worldwide [7]. Notably, the condition is linked to adverse maternal and neonatal effects, such as cesarean delivery, preeclampsia, and fetal macrosomia [8,9]. GDM also has long-term metabolic consequences for women, including an elevated risk of diabetes, dyslipidemia, coronary heart disease, and hypertension [9,10]. Besides that, a 5-year prospective study showed that post-GDM women from South Asia suffered glucose intolerance at a much higher rate than women from other ethnic groups [11]. Kim et al. have reported that the African-American post-GDM women have a ten-fold increased risk of acquiring type 2 diabetes mellitus (T2DM) compared to non-Hispanic white women and Asian/Pacific women [12,13]. With an increased prevalence of T2DM in post-GDM women, it is critical to re-examine the pathophysiology of GDM by adopting a novel approach. The possible use of microbiome knowledge to diagnose and treat GDM is essential in the current situation.

Various microbiome taxa have been reported to be correlated with GDM. For example, increased abundance of *Blautia*, the *Eubacterium hallii* group [14], *Prevotella* [15], *Fusobacterium* [16], *Desulfovibrio*, *Ruminococcus* [17], and *Klebsiella variicola* [18], and reduced abundance of *Akkermansia*, *Parabacteroides*, *Bacteroides* [15], *Anaerosporobacter*, *Marvinbryantia* [17], and *Faecalibacterium* [14,16] in the gut were seen in women with GDM, as opposed to the non-GDM women. Nevertheless, the gut microbiome–GDM association appears inconclusive, primarily due to the heterogeneity in the findings. For example, DiGiulio et al. (2015) reported that the composition of vaginal, distal intestine, and oral microbiota composition and diversity remained generally steady during pregnancy, including in GDM women [19]. On the other hand, several findings discovered microbiota dysbiosis in women with GDM, and it matched the gut microbiota patterns of women with T2DM [17,18,20]. Since the prevalence of T2DM among women with GDM increased [13,21,22], manipulating the gut microbiota during pregnancy may prevent glucose metabolism impairment and improve diabetes-related health outcomes in pregnant women.

Therefore, it is necessary to clarify a more definitive role of identified taxonomic classes in the pathophysiology of GDM. It may pave the route for developing novel biomarkers and therapeutics based on the microbiome. This review aimed to summarize how the gut microbiota modulates the mechanistic pathways involved in GDM pathophysiology.

## 2. Materials and Methods

We conducted a systematic review using the updated Preferred Reporting Items for Systematic reviews and Meta-Analyses (PRISMA) 2009 Statement [23] and checklist (Supplementary Table S1).

### 2.1. Data Sources and Search Strategy

A comprehensive literature search of peer-reviewed articles published in the past ten years was conducted using the following electronic bibliographic databases: Ovid Medline, Scopus, PubMed, CINAPlus, Cochrane Library, Embase, and PsycINFO. The search strategy was built using the following MeSH terms and keywords: ((gut microbiota) OR (microbiota) OR (microbiome)) AND ((motor activity) OR (physical activity) OR (fitness) OR (nutrition) OR (diet) OR (lifestyle) OR (life style) OR (prebiotic) OR (probiotics) OR (anti-diabetic drugs) OR (hypoglycemic drugs)) AND ((gestational diabetes mellitus) OR (gestational diabetes) OR (diabetes in pregnancy)) AND ((gut) OR (fecal) OR (gut flora) OR (dysbiosis) OR (eubiosis) OR (endotoxins) OR (bacteria)) AND ((16S rRNA) OR (metagenomics) OR (sequencing)). Various key words were used to expand the search results and avoid missing potential articles documenting the role of gut microbes in the onset and development of GDM.

We restricted our search to human studies and articles written in English. We also conducted manual searching for the articles using the reference lists of included studies and past reviews.

Supplementary Table S2 shows an example of the search strategy for PubMed conducted on 1 March 2021.

### 2.2. Study Selection

T.K. excluded duplicated studies and screened the titles and abstracts of retrieved references using Endnote Version X9. Subsequently, T.K. and A.R. independently evaluated the full-text eligibility and study selection. Disagreements on article eligibility were resolved through discussion. Studies were included if they met the following criteria: (1) randomized-control trials (RCT) and observational studies conducted among women with GDM; (2) carried out metagenomics sequencing; (3) reported maternal outcomes such as HbAIc, fasting blood glucose level, and gestation weight gain; (4) published between 1 January 2011 to 1 March 2021; and (5) published in the English language. Non-peer-reviewed publications such as book chapters, online abstracts, and conference proceedings were excluded. Studies reporting adults with type 1 or type 2 diabetes mellitus were also excluded.

### 2.3. Data Extraction and Synthesis

T.K. and A.R. independently extracted the following relevant information from included studies using a Microsoft Excel spreadsheet: author, year of publication, country, study design, number of participants, primary and secondary outcomes, measurement tools used, and influencing variables in sequencing. The characteristics of the studies were summarized, and data on the types of gut microbiota and its association with metabolic variables in GDM were qualitatively synthesized.

### 2.4. Quality Assessment and Risk of Bias

The NIH quality assessment tool for observational cohort and cross-sectional and interventional studies was used to assess the included studies’ methodological quality and risk of bias [24]. The quality was assessed based on the study population, eligibility criteria, sample size justification, timeframe to see the effect, exposure, and outcome details, and other sources of bias. Each included study was classified as being of good, fair, or poor quality. All analyses were independently assessed for methodological quality by two reviewers (T.K. and A.R.).

## 3. Results

### 3.1. Study Selection

**Figure 1:**
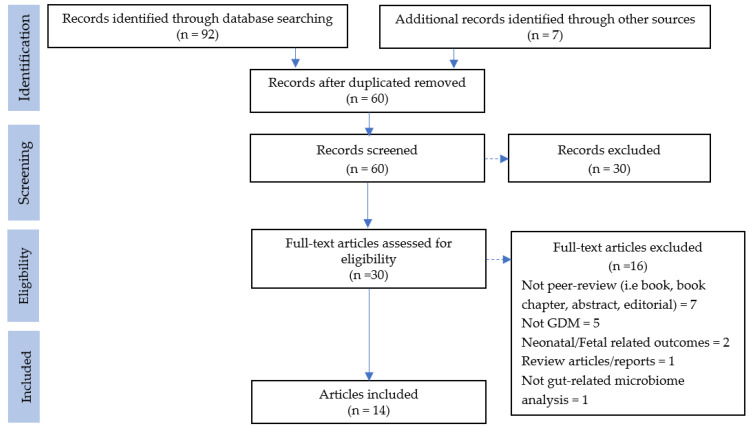
PRISMA flow chart of the article selection process.

The database search resulted in 92 records, while manual searching resulted in an additional seven records. After removing duplicate records (n = 39), 60 titles and abstracts were screened. Subsequently, 30 full texts and their references were screened using the review eligibility criteria. Finally, 14 studies met the inclusion criteria and were included in the systematic review (Figure 1). A summary of all studies included is available as Supplementary Table S3.

### 3.2. Study Characteristics

The study characteristics are summarized in Table 1. Of 14 studies included in this systematic review, 11 studies were observational, three were described as prospective cohorts [17,20,25], two were described as case-controls [26,27], and eight as cross-sectional studies [14,15,16,18,28,29,30,31]. One study was reported as an intervention study [32]. Most of these studies were conducted in China (42.9%) and in Finland (21.4%). All of the included studies had small sample sizes (range 41–150).

### 3.3. Study Quality

The mean score on the NIH Quality Assessment Scale was 50.3% (range 14.3–100%). There were no studies with poor quality; three studies had fair quality and eleven studies had good quality (Supplementary Tables S4–S6).

### 3.4. Modulation of Gut Microbiota in Pregnancy and Its Association with GDM

#### 3.4.1. Distribution of Gut Microbiota during Pregnancy

A summary of the gut microbiome and its metabolic involvement in GDM progression is presented in Table 2. Koren et al. (2012) demonstrated that intestinal microbial distribution evolves dramatically during gestation [25]. During the first trimester, pregnant women’s gut microbe distribution was identical, regardless of GDM status, to that of stable non-pregnant subjects. Conversely, in late pregnancy, the diversity of *Proteobacteria* and *Actinobacteria* increased, whereas that of *Faecalibacterium* decreased. More than 20 different operational taxonomic units (OTUs) were established, with 18 of the OTUs being excessively recorded during the first trimester. Butyrate-producing bacteria such as *Eubacterium* and *Faecalibacterium* trigger the majority of them. The other OTUs, primarily *Enterobaceae* and *Streptococcus*, were highly expressed in late pregnancy. However, across gestational weeks, the relative abundance of Bacteroidetes and Firmicutes was retained. Bacterial diversity (α-diversity) depleted as the third trimester progressed [25].

Moreover, each woman’s microbiota differentiated unexpectedly by the third trimester compared to the first-trimester composition, and it was unrelated to health status and dietary habits. In most women, changes in microbial composition from the first to the third trimester involved a rise in proteobacteria [25] linked to inflammation-related dysbiosis [33]. Samples taken from five healthy pregnant women were implanted into germ-free female mice and analyzed using shotgun metagenomics. The findings showed increased pro-inflammatory cytokines in the germ-free female mice that received third-trimester fecal samples compared to the first trimester’s sample. In addition, the third-trimester fecal sample recipient mice showed evidence of adiposity and increased 30-min postprandial blood glucose levels compared to the first-trimester recipient mice. In conclusion, Koren et al. (2012) revealed that insulin resistance, low-grade inflammation, and adiposity towards the third trimester contributed to intestinal microbial dysbiosis, independent of GDM status in pregnant women [25].

#### 3.4.2. Influences of Gut Microbiota in Obese GDM Women

Several studies have suggested altered microbial diversity throughout gestational weeks, which could be linked to the etiology of GDM [17,18,20,30]. The association between gut microbial dysbiosis in an early gestational week and GDM was reported by Mokkala et al. among overweight women (n = 75) [30]. Fecal samples were collected in the first trimester, and the oral glucose tolerance test (OGTT) conducted at the second trimester found a GDM prevalence of 20% (n = 15). The study found a substantially different total abundance of the Ruminococcaae family among women who developed GDM and non-GDM women. Further analysis found a strong correlation of the Ruminococcaceae family and blood glucose level but not with C-reactive protein (CRP) levels and insulin after controlling for other potential confounders. While there was a limited sample size of GDM women, this research was rigorous, as conflicting variables were strictly excluded.

Interestingly, this finding contrasted to Gao et al. (2020) who reported a negative correlation of several genera from the Ruminococcaceae family with HbA1c in normal BMI women with hyperglycemia in pregnancy [27]. Ruminococcaceae has a role in producing short-chain fatty acids to aid in a positive metabolism [34]. In support of this view, Gomez-Arango et al. (2016) showed that a high Ruminococcaceae abundance during early gestation in overweight and obese women may be associated with poor pregnancy metabolism [27]. Thus, the abundance of the Ruminococcaceae family has been speculated to increase in overweight and obese women with GDM and not in those with normal BMI levels.

A study using lipidomics methods reported a correlation between hyperlipidemia and gut microbes dysbiosis in GDM [31]. *Haemophilus*, *Veillonella*, *Streptococcus*, *Actinomyces*, and *Prevotella*, were found in higher abundance in GDM and hyperlipidemia. Moreover, these microbiotas, except for *Prevotella*, have been linked to higher levels of total cholesterol. Prevosphatidyl glycerol (LPG) and phosphatidylinositol-3 (PIP3) are significantly linked to lipid metabolites associated with obese and diabetic phenotypes [31]. Thus, this information provides compelling evidence of an association with lipid metabolic pathways and the significance of GDM pathogenesis through gut microbial disruption.

Overall, these findings confirmed gut microbiota dysbiosis during early gestational weeks and relevant microbiota prior to GDM diagnosis in overweight and obese women [18,27,29,30,31]. It suggests that gut microbiota modification could be a promising target for GDM prevention in women with high BMI. However, the connection remains to be explained between gut microbiota disturbances and its metabolic pathways to GDM progression among pregnant women.

#### 3.4.3. Association of Gut Microbiota in Glucose Response, Lipid Metabolism, and Inflammation in GDM

Limited studies have analyzed the linkage of metabolic markers to microbial dysbiosis in women with GDM, which has a favorable association with glucose intolerance, adiposity, and low-grade inflammation [15,17,20]. *Faecalibacterium*, for example, was negatively correlated with blood glucose, whereas a lower insulin sensitivity condition was associated with OUTs belonging to *Akkermansia*. Gao et al. (2020) have researched the role of the gut microbial composition in women with hyperglycemia in early pregnancy [27]. They found that HbA1c was positively correlated with the relative abundance of Enterobacteriaceae and Bacteroidaceae families. Kuang et al. (2017) reported discovering pathobionts such as *P. distasonis*, *Catenibacterium Mitsuokai*, and *Klebsiella varicola* in GDM women in the second trimester [18]. Most butyrate-producing bacteria, such as *Methanobrevibacter smithii*, *Alistipess* spp., *Eubacterium* spp., and *Bifidobacterium* spp., were expressed minimally in pregnant women with GDM. In addition, the correlation of gut microbiota disruption and glucose resistance in GDM was also seen in metagenomic linkage groups (MLGs). The distributions of *E. rectale*, *P. distasonis*, and *K. variicola* were different among women with GDM. In addition, functional analyses have found evidence of gut microbiota dysbiosis in women with GDM, which may disrupt the host’s natural metabolic processes. Membrane transport, energy metabolism pathways, and lipopolysaccharide (LPS) pathways were enhanced in the women GDM. It has been suggested that the gut microbiota can accelerate the use of glucose as a form of energy in GDM [18].

Moreover, *Blautia* and the *Eubacterium hallii* group have exhibited substantial changes in the gut microbiota of women with GDM [14,17,31]. *Blautia* and *Eubacterium hallii*, both members of the Lachnospiraceae family, play a role in the progression of diabetes [35,36]. *Blautia* was found in higher abundance in people having glucose intolerance [37] and was linked to metabolites, indicating an unfavorable metabolic state in overweight people [38]. On the other hand, *Sutterella*, *Bacteroides*, the Fusobacteriaceae family, and the *Fusobacterium* genus were linked to inflammation markers such as hs-CRP and CRP levels [14,17,20,27]. CRP is mainly used as a marker of inflammation and is associated with diabetes. However, none of these findings examine pro-inflammatory markers to substantiate the relationship between gut microbial disruption and the risk of inflammation in women GDM.

The findings of phyla Firmicutes and Bacteroidetes abundance and their ratio is also scarce among GDM women. Wang et al. examined the gut microbiome of expectant mothers and compared them with oral and vaginal microbiota regions [16]. The results showed that the phylum Firmicutes in intestinal bacterial diversity were identical in GDM and healthy pregnant women. Similarly, Crusell et al. [17] observed that the phyla Firmicutes and Bacteroidetes were prevalent in GDM and non-GDM women. However, they did not assess or compare the ratio of these phyla among the individuals. Cortez et al. (2019) have demonstrated the Firmicutes/Bacteroidetes ratio to be high in women with GDM compared to the control [15]. Ferrocino et al. (2018) concluded that GDM women who follow the recommended dietary guide throughout the gestation weeks have a significantly low level of *Bacteroides*, an increase in Firmicutes, and improvement in the metabolic profile compared to the non-adherent GDM women [20].

#### 3.4.4. Gut Microbiota Pattern in Post-Pregnancy Women with a History of GDM

Three studies have been based on the gut microbiome diversity among women with a previous history of GDM [17,25,28]. The reassessment of gut microbiota confirmed that depleted gut microbiota across pregnancy remains consistent even at postpartum [25]. However, the distribution of gut microbiota at post-pregnancy was found to vary according to studies. Crusell et al., for example, reported Actinobacteria and Firmicutes phyla to be the most common group of microbiota among women with a history of GDM [17]. In contrast, another study by Fugmann et al. (2015) reported that relative Firmicutes phylum abundance was slightly reduced in women who had GDM compared to the women with healthy pregnancies [28]. In the post-GDM women’s subgroup, the proportion of Prevotellaceae was slightly higher at the family level. However, both study groups had equal levels of bacterial diversity (α-diversity) [28].

As an overall finding, research into GDM revealed a wide variety of gut microbiota dysbiosis linked to increased pathobionts from the Bacteroidetes, Proteobacteria, Firmicutes, and Actinobacteria phyla, such as *Desulfovibrio*, Ruminococcaceae, *P. distasonis*, Enterobacteriaceae, *Collinsella*, and *Prevotella*. However, *Bifidobacterium* and *Faecalibacterium*, which produce butyrate, are depleted. Inflammation, adiposity, and glucose intolerance are all linked to gut microbiota dysbiosis in women with GDM. Moreover, those gut microbiota profiles appear similar to those in T2DM subjects. The disrupted gut microbial profile of women with GDM persists even after the delivery, suggesting that it could be used as a T2DM biomarker. Despite this, most of these findings failed to find a clear linkage between the gut microbiota and GDM development.

## 4. Discussion

Several metabolic changes occur during pregnancy, promoting adipose tissue formation in the early months and boosting insulin resistance and lipolysis as pregnancy progresses. Pregnancy-induced insulin resistance leads to an increase in postprandial free fatty acids, hepatic glucose synthesis, and severe insulin resistance [39,40].

Studies have demonstrated that the intestinal microbiota contributes to the development of pre-diabetic disorders such as insulin resistance. It has been found that those with elevated insulin resistance have a significantly altered microbiota, with an unusually high ratio of Firmicutes/Bacteroidetes versus healthy adults [41,42,43].

Research conducted by Fugmann et al. (2015) examined the gut microbiota compositions of women who had developed GDM and women who had normal blood sugar levels 3 to 16 months after giving birth [28]. Although alpha and beta diversity did not change between both the groups, the Firmicutes phylum was reduced in the women with GDM. Serino et al. (2013) also found a link between insulin activity and gut microbiota composition [41]. According to a study by Koren et al. (2012) the gut microbiota composition varies with increasing gestational age in healthy pregnant women and GDM [25]. However, the gut microbiota diversity was decreased in the first trimester in women who eventually developed GDM. According to the study’s findings, women with greater insulin resistance and glucose levels and obesity had more inflammation markers in their stools during the first and third trimester of the pregnancy.

Thus far, several studies have linked gut microbial composition to insulin resistance. The structure of the microbiome pattern in GDM appeared to be comparable to T2DM and metabolic disorders [17,18,20]. However, the bacterial profile of pregnant women is influenced by ethnicity, environment, dietary intake, BMI, genetics, and antibiotic use. Thus, the link between GDM and gut microbiome must be further explored. The patterns of unique taxa and their functionality are highlighted in the Results section (Table 2). The links between these gut microbiota patterns and the main mechanisms involved in GDM development are summarized below.

### 4.1. Microbiota Impact on Metabolism in Women with GDM: Potential Pathways

#### 4.1.1. Modulation of Inflammation

The Gram-negative pathobiont *Alistipes* was shown to be associated with women with GDM who followed a high-fat diet (HFD) [20]. In other trials, *Sutterella*, *Parabacteroides*, *Prevotella, E. coli*, and *Desulfovibrio*, categorized under Gram-negative pathobionts, were consistently elevated in women with GDM [17,18,25,31]. These bacteria generate lactate or butyrate, modulating gut integrity and triggering an inflammatory response in the gut that promotes diabetes development [44,45]. There is also a substantial increase in pathways linked to LPS synthesis and its export system in women with GDM [18,20,25]. LPS is a component of Gram-negative bacteria’s cell walls that can trigger an inflammatory reaction [46,47]. The decreased Gram-positive bacteria in women with GDM included *Clostridium* species that lack LPS [18,25]. The increased LPS process and imbalance of Gram-negative and Gram-positive pathobionts in women with GDM may have compromised the gut permeability. This whole process allows LPS to cross the gut epithelial layer and into the systemic circulation. It can lead to metabolic endotoxemia and inflammatory response activation, resulting in low-grade inflammation. Thus, the enriched Gram-negative pathobiont in women with GDM may enhance low-grade inflammation, impaired insulin signaling, and plasma glucose regulation, resulting in a hyperglycemia condition.

#### 4.1.2. Glucose Metabolism

Obesity-induced resistance to insulin is another suggested pathway. The effects of dietary consumption and gut microbes on glucose metabolism during the second and third trimesters were studied in overweight women with GDM [20]. Firmicutes appeared to be higher in level, whereas Actinobacteria and Bacteroidetes were minimal in GDM participants who consumed less fiber and more sugar and fat [20]. Crusell et al. (2018) also have reported that the Firmicutes phylum is predominant in GDM women [17]. An imbalance in the Firmicutes/Bacteroidetes ratio has already been related to dysbiosis conditions [48,49]. Firmicutes and Bacteroidetes potentially mediate insulin resistance by modulating glucagon-like peptide 1 (GLP-1) secretion [12,50]. GLP-1 is a gut hormone that L-cells primarily release in response to nutritional status [51,52]. The active form of GLP-1 (7–36 amide) regulates satiety in the brain, insulin release in pancreatic islets, and glucose absorption in muscle to maintain systemic energy homeostasis [53]. Moreover, Ruminococcaceae was associated with pregnancy-induced insulin resistance in obese women, and it is possible that the bacteria altered insulin signaling impairment of glucose homeostasis and inflammation [29,30].

In addition, metabolic pathways such as gluconeogenesis/glycolysis, galactose metabolism, and sucrose metabolism were disturbed in women with GDM [20]. Respondents who did not adhere to the dietary recommendations had higher weight increase during gestation, high lipid profile levels, high CRP level, insulin resistance, and poor glycemic control. It suggests a potential linkage between nutrition, gut microflora, metabolic processes, glucose intolerance, and low-grade inflammation in women with GDM [20]. The involvement of short-chain fatty acids (SCFAs) in the metabolic process and low-grade inflammation is one mechanism that could be studied to understand how dietary intake and gut microbes can affect insulin resistance and obesity in pregnant women.

#### 4.1.3. Fatty Acid Oxidation, Synthesis, and Energy Expenditure

**Figure 2:**
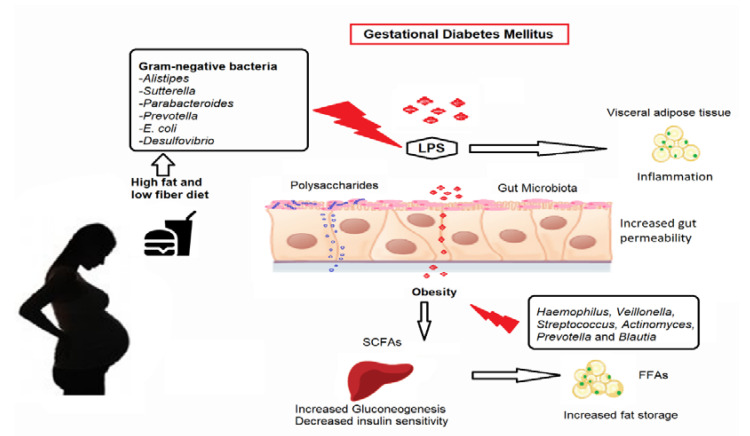
Potential mechanisms of pathobiont adherence and efflux through the gut epithelium in GDM women. LPS, lipopolysaccharides; FFAs, free fatty acids; SCFAs, short-chain fatty acids. Note: Images were obtained separately from Microsoft image search as licensed by the Creative Commons License (CC BY 2.0) and edited in Microsoft Paint. Poor adherence to recommended daily dietary intake, such as an increase in high-fat/low-fiber diet consumption, may have altered the makeup of the normal gut microbiota. It increased the Gram-negative pathobionts and SCFAs. The presence of Gram-negative pathobionts may have raised LPS biosynthesis levels. The increased gut permeability condition permits pathobionts, LPS, and SCFAs to move across the epithelial layer of the gut. The crossed LPS, SCFAs, and pathobionts entered the systemic circulation and reached peripheral tissues. Excessive SCFAs may have increased gluconeogenesis in the liver and elevated plasma glucose levels. In addition, SCFAs might have enhanced FFAs uptake and increased lipogenesis, causing excessive fat storage with the aid of other pathobionts in overweight pregnant women. LPS causes metabolic endotoxemia and inflammatory response activation, resulting in low-grade inflammation and adiposity. These mechanisms lead to glucose intolerance in GDM women.

In women with GDM, imbalanced dietary intake with high-fat and low-fiber nutrition may have changed the normal gut microbial composition. It causes an increase in bacteria producing butyrate, including Firmicutes and *Faecalibacterium*, and eventually leads to excessive SCFA activity. SCFA levels may have surpassed the adipose tissue’s natural lipid storage capability instead of energy expenditure, resulting in a good energy balance. However, an overabundance of free fatty acids in the systemic circulation leads to a rise in lipid storage in the skeletal muscle and liver, and hence obesity conditions develop. Furthermore, by upregulating pro-inflammatory markers, unneeded SCFAs can develop low-grade inflammation. Hyperglycemia in women with GDM can be caused by the increased mechanisms of glycolysis/gluconeogenesis and by the inhibition of insulin signaling in peripheral tissues. This concept is supported by the fact that women with GDM have a higher carbohydrate metabolism (glycolysis/gluconeogenesis) and a lower fatty acid metabolism [20]. These three potential mechanistic pathways, and the structure of microbiota pattern and functionality are illustrated in Figure 2.

### 4.2. Challenges in Gut Microbiota Research

While numerous studies in women with GDM has been conducted on the gut microbiota composition, the results remain conflicting. The nature of research, the geographical locations, the total sample size, the participant registration restrictions, gestational age at the end of fecal samples collection, and the sequencing methodologies were variable, leading to variances in outcomes.

#### 4.2.1. Study Design

A prospective cohort research design is recommended since it makes it possible to determine causal links such as the association of gut microbiota with GDM in this case. Most of the published studies, however, used a cross-sectional approach (Table 1). Furthermore, most of the studies were conducted at different geographical places, resulting in ethnic and dietary intake variations in the results.

Most studies in this systematic review (71.4%) used international diagnostic methods from the International Association of the Diabetes and Pregnancy Study Groups (IADPSG) and the American Diabetes Association (ADA) (Supplementary Table S3) to diagnose GDM. This was the single step (2 h-75 g OGTT) benchmark values of fasting ≥ 5.1 mmol/L, 1 h ≥ 10.0 mmol/L, and 2 h ≥ 8.5 mmol/L. However, three studies have not reported the diagnostic criteria used in their study design [16,17,25]. The lack of international agreement in screening and diagnostic criteria for GDM often leads to variations in GDM prevalence among countries in the world [9], which could affect the interpretation of microbial findings. For example, countries that adopt low sensitivity diagnostic methods (e.g., reducing the two-hour threshold value of OGTT) may fail to identify women who are at risk of metabolic outcomes that lead to ‘missing out’ in total abundance and type of microbiota. In contrast, the use of overly sensitive diagnostic criteria could misclassify cases of GDM. Non-GDM women may be assigned to the GDM group, which may mask the variance in gut microbiota abundance and types between the two groups. One possible way to avoid this conflict is to classify the groups according to severity of GDM level such as “only diet modification group”, “insulin group”, “successful treatment group”, and “failure of treatment group”. This step may provide us more precise findings in terms of the abundance and types of microbiota among the GDM populations.

#### 4.2.2. Sample Size

The sample size is essential because the relevance of the results can be affected and it is vital for the clinical application of the results. Most of the papers included did not justify the sample size merely due to the observational research. However, prospective cohort studies similarly failed to justify the sample size included in their research [17,20,25]. A limited number of studies had small sample sizes with less than 30 per group [14,31]. A few studies had enrolled a differing number of participants in groups with and without GDM (Supplementary Table S3).

#### 4.2.3. Enrollment Criteria

The majority of the subjects were overweight, and they are above 35 years old. Numerous researches have shown that women with GDM have a higher prepregnancy BMI than healthy women [15,27] (Supplementary Table S3). However, a few studies have shown that these variables were modified to reduce the impact of potential confounders [17,20,30].

#### 4.2.4. Time of Sample Collection

The timing of fecal selection, which was inadequate in most studies, was another crucial consideration. The disturbance of gut microbes is reported to have started in the third trimester of pregnancy [25]. However, the majority of research only took feces once over the gestational weeks. Moreover, around 50% of studies collected feces in the late trimesters, while others only obtained fecal samples in the first or second trimester, and postpartum. Minimal research has distinguished the characteristics of the gut microorganisms during and after the pregnancy (Table 1). This could lead to various erroneous findings.

#### 4.2.5. Methods of Sampling and Sequencing Tool

The sampling method chosen (Table 3) may have played a role in the differences in the outcomes between the samples. Pathobiont adherence and some host metabolisms have previously existed in the small intestine [54,55]. As a result, taking an intestinal region sample is better for determining the connection between GDM and gut microbes’ composition. However, since it is an invasive, expensive, and time-consuming process, the feasibility may be challenging. In addition, it is crucial to choose the right sequencing tool to make comparisons with other studies easier. The 16S sequencing technique was used in the majority of the published trials, which used various regions. The choice of a suitable area, amplicon primer configuration, and amplification phase are critical since they may introduce biases and lead to contradictory findings [56,57]. For example, Crusell et al. (2018) amplified the V1–V2 regions using a 27F/338R primer [17], while Wang et al. (2018) used updated 342F and 805R primers to amplify the V3–V4 regions [16]. Although 16S sequencing is a more cost-effective and reliable process, the study was limited by the type of bacteria. As a result, it was unable to detect the gut microbiota compositions at lower taxonomic stages. The high expression level of *Faecalibacterium*, an anti-inflammatory bacterium, showing a favorable association with the inflammatory marker hs-CRP, is an example of a contradictory finding [17]. The researchers speculated that the function of *Faecalibacterium* could be specific strain makeup, and future analysis is needed to justify the discrepancies through lower taxonomic level detections with the advanced shotgun metagenomics sequencing methods.

Two studies used shotgun metagenomic sequencing to determine the makeup of the gut microbes in women with GDM [18,32]. Kuang et al. (2017) managed to identify gut microbiota disruption at lower taxonomic levels in normal-weight women with GDM and discovered a large amount of MLGs that varied between GDM and non-GDM women from a single time sample collection [18]. In addition, some pathways linked to LPS processes and energy metabolism were enhanced, whereas insulin signaling pathways were decreased in women with GDM compared to control subjects. However, Mokkala et al. (2021) reported that neither specific gut microbiota species nor their role is involved in the onset of GDM in overweight and obese women [32]. They collected samples at two different time points from the participants. Therefore, the differences in study population characteristics and frequency of stool sample collection led to the variations of results among these two studies.

To validate the association between gut microbes and GDM, potential major prospective trials with more than one time of fecal samplings, usage of high-tech sequencing platforms, appropriate evaluation, and elimination of conflicting variables are needed. There are two limitations to this review. Firstly, limiting the review to English language papers may have excluded papers published in other languages, thus possibly missing pertinent information. Secondly, no gray literature was included. Despite these limitations, we could retrieve literature from the past decade using broad search terms and seven bibliographic databases that gave strength to our systematic review.

### 4.3. Future Recommendation

Given that GDM is a complicated disease, it is challenging to distinguish pathogenic bacteria from the gut microbiome. In human studies, predisposing variables such as geographic place, ethnicity, health condition, and medication use cause ambiguity in identifying pathogenic bacteria linked with GDM. Nonetheless, since extracting from the human intestine is difficult, most researchers use stool samples for microbiome studies. The microbial profile from the feces does not entirely reflect the gut microbiome. Moreover, substantial research has been based on genomic information, with rare transcriptome, proteome, and metabolome studies. The vast majority of the human interaction studies make no effort to identify pathogens that might influence and/or have a causal role in GDM development, which appears to be a key topic in the field. Although suggesting causative relationships in associated bacteria is challenging, high-tech systems may aid the researchers. Innovative methods such as Transkingdom Network Analysis [58] and revolutionary Mendelian Randomization methods [59] for determining which microorganisms and microbial genetic makeup regulate host biological activities have currently been designed and administered.

In addition, rigorous studies need to be conducted to establish novel treatment, preventive, and clinical microbiota instruments targeting GDM. To begin, while identifying medical conditions for research, microbiome studies must account for the pharmacological, cellular, and genetic variation of GDM women, and the pharmacogenomics profiles derived from individual treatment reactions with anti-diabetic medications or insulin prescription. While the incidence of T2DM has been correlated with gut microbial patterns in published literature, we are unable to explore the association between microbial fingerprinting with GDM due to lack of relevant studies. We also found a lack of studies exploring the obese host–gut microbiota interactions in GDM. These are potential research gaps that can be fulfilled in future studies.

Finally, since feces constitute only a tiny portion of the gut microbiota, non-invasive techniques for extracting gut bacteria samples from various areas anywhere along the digestive tract are necessary.

## 5. Conclusions

Even though several studies have demonstrated the role of gut microbe composition in GDM pathophysiology, the field is still in its infancy. Currently, we believe that specific microbial taxa and associated molecular pathways are involved in glucose metabolism in GDM. However, because of the heterogeneity of GDM and the redundancy of the gut microbiota, high-tech treatment (e.g., fecal transplant) is not guaranteed. On the other hand, we should strive for precision/personalized medicine, in which diabetes medication and probiotics are prescribed for a specific patient depending on the synthesis and interacting nature with their microbial genomes. Pregnant women who are at risk of GDM are also recommended to increase fiber-rich foods and reduce the intake of high fats to prevent the modulation of normal gut microbiota that can increase the risk of the disease.

## Figures and Tables

**Table 1:** Summary characteristics of included studies (n = 14).

Characteristics		n (%)
Study design	Cross-sectional	8 (57.1)
	Prospective cohort	3 (21.4)
	Case-control	2 (14.2)
	Intervention	1 (7.4)
Country	China	6 (42.9)
	Finland	3 (21.4)
	Germany	1 (7.4)
	Australia	1 (7.4)
	Denmark	1 (7.4)
	Brazil	1 (7.4)
	Italy	1 (7.4)
Sample size	<50	2 (14.2)
	50–100	7 (50.0)
	>100	5 (35.8)
Gestational age (trimester)	First	3 (21.4)
	Second	3 (21.4)
	Third	3 (21.4)
	Post-partum	1 (7.4)
	Multiple	4 (26.6)
Body weight	Overweight/obese	7 (58.3)
	Normal weight	5 (41.7)

**Table 2:** Types of gut microbiota and their impact on the metabolic variables in GDM.

Gut Microbiome	Metabolic Outcome																	References
	BG	Insulin	HbA1c	BMI	Adiposity	HDL	Leptin	TG	Adipokine	TC	PG	LPEt	LdMePE	LPS	CRP	GI	PC	
Proteobacteria	↑				↑			↑		↑							↑	[25,31]
Actinobacteria	↑				↑												↑	[25]
*Faecalibacterium*	↓↑				↓			↑			↑	↑	↑				↓	[16,20,25,31]
Firmicutes	↑			↓		↓	↓											[15,28]
Bacteroidaceae			↑	↓		↓	↓							↑	↑			[20,27,28]
Prevotellaceae	↑		↑	↑		↑	↑											[17,20,28]
Ruminococcaceae	↑								↑									[29,30]
Lachnospiraceae	↑								↑									[18,29]
*Collinsella*		↑																[20,29]
*Holdemania filiformis*	↑																	[33]
*Eisenbergiella*	↑																	[26]
*Tyzzerella 4ycer*	↑																	[26]
*Haemophilus*										↑								[31]
*Veillonella*										↑								[31]
*Actinomyces*										↑								[31]
*Streptococcus*										↑								[31]
*Christensenella*	↑																	[17]
*Akkermansia*		↓																[17]
*Blautia*	↑		↑															[14,17,20]
*Sutterella*														↑	↑			[20]
*Alistipes*					↑													[20]
*Allofustis seminis*	↑																	[18]
*Megamonas* spp.	↑																	[18]
*Eggerthella* spp.	↑																	[18]
*E. rectale*	↑																	[18]
*K. variicola*	↑																	[18]
*P. distasonis*	↑																	[18]
*Coprococcus*																↑		[29]
*Eubacterium_hallii*	↑																	[14]
Enterobacteriaceae			↑															[27]
Fusobacteriaceae															↑			[27]

Note: ↑ = increased abudance; ↓ = decreased abudance. BG (blood glucose); TG (triacylglycerols); TC (total cholesterol); PG (phosphatidylglycerols); LPEt (lysophosphatidylethanol); LdMePE (dimethylphosphatidylethanolamine); LPS (lipopolysaccharide) biosynthesis; GIP (incretin hormone glucose-dependent insulinotropic polypeptide); PC (pro-inflammatory cytokines).

**Table 3:** Methodological characteristics of included studies (n = 14).

Characteristics		n (%)
Sample	Fecal	14 (100.0)
Immediate storage temperature	4 °C	1 (8.3)
	−20 °C	6 (50.0)
	−80 °C	5 (41.7)
DNA isolation methods	QIAamp DNA Stool Mini Kit	8 (61.5)
	PSP Spin Stool DNA Plus Kit	1 (7.7)
	NucleoSpin Soil kit (Macherey-Nagel	1 (7.7)
	RNeasy Power Microbiome KIT	1 (7.7)
	PowerMax (stool/soil) DNA isolation kit	1 (7.7)
	GTX stool extraction kit	1 (7.7)
Sequencing	Amplicon	12 (85.7)
	Metagenomic	2 (14.3)
Variable region amplified	V1–V2	2 (18.1)
	V3–V4	5 (45.5)
	V4	3 (27.3)
	V6–V8	1 (9.1)
Platform	Illumina HiSeq	5 (45.5)
	Illumina MiSeq	8 (61.5)
	Unknown	1 (9.1)
Bioinformatics pipeline	RDP classifier	1 (7.14)
	QIIME	8 (57.1)
	MOCAT	1 (7.14)
	Multiple	4 (28.6)
Reference database	Silva	6 (50.0)
	Greengenes	4 (33.0)
	Vsearch	1 (8.3)
	EzTaxon	1 (8.3)

## Data Availability

Data sharing not applicable.

## Supplementary Materials

The following are available online at https://www.mdpi.com/article/10.3390/biology10101027/s1, Table S1: PRISMA 2009 Checklist; Table S2: Search strategy in PubMed; Table S3: Summary of the included studies; Table S4: Quality assessment of the prospective cohorts and cross-sectional studies (n = 11); Table S5: Quality assessment of case-control studies (n = 2), Table S6: Quality assessment of included intervention (n = 1).
