# Supplementary material for: The Modulation of Gut Microbiota Composition in the Pathophysiology of Gestational Diabetes Mellitus: A Systematic Review
Source: Biology (Basel). 2021 Oct 11;10(10):1027. doi: 10.3390/biology10101027 (PMC8533096; doi:10.3390/biology10101027)
Supplement: Supplementary file 1 [file biology-10-01027-s001.zip › biology-1400868-supplementary.pdf]

Supplementary Table S1. PRISMA 2009 Checklist

Section/topic	#	Checklist item	Reported on page #
**TITLE**			
Title	1	Identify the report as a systematic review, meta-analysis, or both.	Page 1
**ABSTRACT**			
Structured summary	2	Provide a structured summary including, as applicable: background; objectives; data sources; study eligibility criteria, participants, and interventions; study appraisal and synthesis methods; results; limitations; conclusions and implications of key findings; systematic review registration number.	Page 1
**INTRODUCTION**			
Rationale	3	Describe the rationale for the review in the context of what is already known.	Page 1-2
Objectives	4	Provide an explicit statement of questions being addressed with reference to participants, interventions, comparisons, outcomes, and study design (PICOS).	Page 2
**METHODS**			
Protocol and registration	5	Indicate if a review protocol exists, if and where it can be accessed (e.g., Web address), and, if available, provide registration information including registration number.	n/a
Eligibility criteria	6	Specify study characteristics (e.g., PICOS, length of follow-up) and report characteristics (e.g., years considered, language, publication status) used as criteria for eligibility, giving rationale.	Page 3
Information sources	7	Describe all information sources (e.g., databases with dates of coverage, contact with study authors to identify additional studies) in the search and date last searched.	Page 2
Search	8	Present full electronic search strategy for at least one database, including any limits used, such that it could be repeated.	Supplementary Table S2
Study selection	9	State the process for selecting studies (i.e., screening, eligibility, included in systematic review, and, if applicable, included in the meta-analysis).	Page 3
Data collection process	10	Describe method of data extraction from reports (e.g., piloted forms, independently, in duplicate) and any processes for obtaining and confirming data from investigators.	Page 3
Data items	11	List and define all variables for which data were sought (e.g., PICOS, funding sources) and any assumptions and simplifications made.	Page 3
Risk of bias in individual studies	12	Describe methods used for assessing risk of bias of individual studies (including specification of whether this was done at the study or outcome level), and how this information is to be used in any data synthesis.	Page 3
Summary measures	13	State the principal summary measures (e.g., risk ratio, difference in means).	n/a
Synthesis of results	14	Describe the methods of handling data and combining results of studies, if done, including measures of consistency (e.g., I^2) for each meta-analysis.	n/a

Risk of bias across studies	15	Specify any assessment of risk of bias that may affect the cumulative evidence (e.g., publication bias, selective reporting within studies).	n/a
Additional analyses	16	Describe methods of additional analyses (e.g., sensitivity or subgroup analyses, meta-regression), if done, indicating which were pre-specified.	n/a
**RESULTS**			
Study selection	17	Give numbers of studies screened, assessed for eligibility, and included in the review, with reasons for exclusions at each stage, ideally with a flow diagram.	Page 3, Figure 1
Study characteristics	18	For each study, present characteristics for which data were extracted (e.g., study size, PICOS, follow-up period) and provide the citations.	Supplementary Table 3
Risk of bias within studies	19	Present data on risk of bias of each study and, if available, any outcome level assessment (see item 12).	Supplementary Table 4-6
Results of individual studies	20	For all outcomes considered (benefits or harms), present, for each study: (a) simple summary data for each intervention group (b) effect estimates and confidence intervals, ideally with a forest plot.	Table 1-3
Synthesis of results	21	Present results of each meta-analysis done, including confidence intervals and measures of consistency.	n/a
Risk of bias across studies	22	Present results of any assessment of risk of bias across studies (see Item 15).	n/a
Additional analysis	23	Give results of additional analyses, if done (e.g., sensitivity or subgroup analyses, meta-regression [see Item 16]).	n/a
**DISCUSSION**			
Summary of evidence	24	Summarize the main findings including the strength of evidence for each main outcome; consider their relevance to key groups (e.g., healthcare providers, users, and policy makers).	Figure 2, Page 5-14
Limitations	25	Discuss limitations at study and outcome level (e.g., risk of bias), and at review-level (e.g., incomplete retrieval of identified research, reporting bias).	Page 13
Conclusions	26	Provide a general interpretation of the results in the context of other evidence, and implications for future research.	Page 14
**FUNDING**			
Funding	27	Describe sources of funding for the systematic review and other support (e.g., supply of data); role of funders for the systematic review.	Page 15

From: Moher D, Liberati A, Tetzlaff J, Altman DG, The PRISMA Group (2009). Preferred Reporting Items for Systematic Reviews and Meta-Analyses: The PRISMA Statement. PLoS Med 6(6): e1000097. doi:10.1371/journal.pmed1000097

For more information, visit: **www.prisma-statement.org**.

Supplementary Table S2. Search strategy in PubMed database*

#No	Query	Results
5	(((((gut microbiota) OR (microbiota) OR (microbiome)) AND ((motor activity) OR (physical activity) OR (fitness) OR (nutrition) OR (diet) OR (lifestyle) OR (life style) OR (prebiotic) OR (probiotics) OR (anti-diabetic drugs) OR (hypoglycemic drugs))) AND ((gestational diabetes mellitus) OR (gestational diabetes) OR (diabetes in pregnancy))) AND ((gut) OR (faecal) OR (gut flora) OR (dysbiosis) OR (eubiosis) OR (endotoxins) OR (bacteria))) AND ((16S rRNA) OR (metagenomics) OR (sequencing))) Filters: in the last 10 years	30
4	(16S rRNA) OR (metagenomics) OR (sequencing)	2,033,995
3	(gut) OR (faecal) OR (gut flora) OR (dysbiosis) OR (eubiosis) OR (endotoxins) OR (bacteria)	2,651,122<br>4
2	(gestational diabetes mellitus) OR (gestational diabetes) OR (diabetes in pregnancy)	43,884
1	(motor activity) OR (physical activity) OR (fitness) OR (nutrition) OR (diet) OR (lifestyle) OR (lifestyle) OR (prebiotic) OR (probiotics) OR (anti-diabetic drugs) OR (hypoglycemic drugs)	2,048,103

*As of 1 March 2021. Comparable strategy was used in other databases.

Supplementary Table S3. Summary of included studies (n=14)

Study, country	Study design, n, Mean age (SD) (years)	Time-point	Mean BMI (SD) (kg/m²)	Methodology			Main findings
				Sample, storage	DNA isolation method, sequencing, Variable region amplified	Platform, Bioinformatics pipeline, Reference database	
Koren (2012) [25]<br><br>Finland	Prospective cohort<br><br>GDM (n=15)<br>Non-GDM (n=76)<br><br>NA	1st time point at 13 weeks, 2nd time point at 33 weeks and 3rd time point at 1-mo post-partum	overall have normal pre-pregnancy weight: 18.5 to 24.9	Fecal<br><br>Stored at -20⁰C	QIAamp DNA Mini Kit<br><br>16S rRNA<br><br>V1-V2	Illumina HiSeq 2000<br><br>QIIME<br><br>Greengenes	Gut microbiota changed dramatically from first (T1) to third (T3) trimesters, with vast expansion of diversity between mothers, an overall increase in *Proteobacteria* and *Actinobacteria* and reduced richness.
Fugmann (2015) [28]<br><br>Germany	Cross-sectional<br><br>Post-GDM (n = 42)<br>Women after a normoglycemic pregnancy (n = 35)<br><br>Post-GDM:37 [34–39]<br>Normal: 36 [32–38]	Once between 3 to 16 months after post pregnancy	Post-GDM: 27.0 (23.9–31.6);<br><br>Normal: 22.6 (21.3–26.2)	Fecal<br><br>Stored at -80⁰C	PSP Spin Stool DNA Plus Kit<br><br>16S rRNA<br><br>V4	Illumina MiSeq<br><br>UCHIME, RDP classifier<br><br>EzTaxon	Women with a *Prevotellaceae*-dominated gut microbiome were overrepresented in the previous GDM group ($p < 0.0001$). The relative abundance of the phylum *Firmicutes* was significantly lower in women previous GDM (median 48.5 vs. 56.8%; $p = 0.013$).
Gomez-Arango (2016) [29]<br><br>Australia	Cross-sectional<br><br>GDM (n=26)<br>Control (n=44)	Once between 10-15 weeks	Overweight: 26.10<br><br>Obese: 35.60	Fecal<br><br>Stored within one day after collection at -80°C.	QIAGEN AllPrep DNA extraction kit<br><br>16S rRNA<br><br>V6-V8	Illumina MiSeq<br><br>QIIME<br><br>Greengenes	Adipokine levels were strongly correlated with *Ruminococcaceae* and *Lachnospiraceae*. Insulin was positively correlated with the genus *Collinsella*. Gastric inhibitory polypeptide was

							positively correlated with the genus *Coprococcus* but negatively with family *Ruminococcaceae.*
Kuang (2017) [18]<br><br>China	Cross-sectional<br><br>GDM (n=43)<br>Control (n=81)<br><br>NA	Once between 21-29 weeks	NA	Fecal<br><br>Frozen at -20°C freezers immediately (within 30 minutes) and transferred to -80°C	QIAamp DNA Stool Mini Kit<br><br>Whole-metagenome shotgun<br><br>NA	Illumina HiSeq 2500<br><br>MOCAT<br><br>NCBI and BLASTN	*Parabacteroides distasonis*, *Klebsiella variicola*, were enriched in GDM patients, whereas *Methanobrevibacter smithii*, *Alistipes* spp., *Bifidobacterium* spp. and *Eubacterium* spp. were enriched in controls.
Mokkala (2017) [30]<br><br>Finland	Cross-sectional<br><br>GDM (n=15)<br>Control (n=60)<br><br>GDM:29.3 (3.2);<br>Normal: 30.3 (4.6)	Once at average 12.9 weeks	Pregnancy BMI GDM: 29.8 (3.5); Normal: 30.0 (4.5)	Fecal<br><br>NA	NA<br><br>16S rRNA<br><br>NA	NA<br><br>QIIME<br><br>NA	OR for a percent point increase in *Ruminococcaceae* was 1.12 (95% CI 1.02–1.2; adjusted for BMI, intervention group, gestational weeks, and fat and fibre intake, P = 0.017). A statistically significant correlation was detected between *Ruminococcaceae* and glucose (Pearson correlation, ϱ = 0.31, P = 0.007) but not with hs-CRP (ϱ = −0.150, P = 0.20) or insulin (ϱ = 0.09, P = 0.45).
Crusell (2018) [17]<br><br>Denmark	Prospective cohort<br><br>GDM (n=50)<br>Control (n=157)<br><br>GDM: 33.8 (4.6)<br>Control: 34.4 (4.4)	1st time point at third trimester and 2nd time point at 8-mo postpartum	GDM: 29.3 (5.6)<br>Control: 27.1 (4.8)	Fecal<br><br>Stored in -18^{0}C, transferred to laboratory within	NucleoSpin Soil kit (Macherey-Nagel<br>16S rRNA<br><br>V1-V2	Illumina MiSeq<br><br>RDP classifier<br><br>Vsearch	*Actinobacteria* at phylum level and *Collinsella*, *Rothia* and *Desulfovibrio* at genus level had a higher abundance in the GDM cohort. Negative association between *Butyricicoccus* and insulin

				48hours and subsequently at -80⁰C.			sensitivity (r = − 0.12; Q = 0.08) and positive correlations between stimulated 2-h plasma glucose level and *Prevotella* (r = 0.25; Q = 0.03) and *Faecalitalea* (r = 0.26; Q = 0.03), after BMI adjustment.
Cortez (2018) [15]<br><br>Brazil	Cross-sectional<br><br>GDM (n=26)<br>Non=GDM (n=42)<br><br>35.1 (3.8)	Once in third trimester (28–36) weeks	NA	Fecal<br><br>Stored in freezer, and subsequently at -80⁰C in the laboratory.	QiaAmp DNA Stool Mini Kit<br><br>16S rRNA<br><br>V4	Illumina MiSeq<br><br>QIIME<br><br>Silva	*Bacteroides* was higher in the control group in comparison to GDM patients whereas *Ruminococcus*, *Eubacterium*, and *Prevotella* were higher in the GDM group. However, none of these differences were statistically significant (p > 0.01).
Ferrocino (2018) [20]<br><br>Italy	Prospective cohort<br><br>41 GDM<br><br>37.1 (4.2)	Once between 24-28 weeks and at 38 weeks	Pre-pregnancy: 25.8 (5.9); Study start: 28.2 (5.3); Study end: 29.4 (5.4)	Fecal<br><br>Stored immediately at 4⁰C, transported to laboratory within 12 hours at refridgerated temperature, subsequently storedat -80⁰C	RNeasy Power Microbiome KIT (Qiagen, Milan, Italy)<br><br>16S rRNA<br><br>V3-V4	Illumina MiSeq<br><br>QIIME<br><br>Greengenes	*Faecalibacterium* was significantly associated with fasting glucose; *Collinsella* (directly) and *Blautia* (inversely) with insulin, and with HOMA-IR, while *Sutterella* with CRP levels.
Wang (2018) [16]	Cross-sectional<br><br>GDM (n=74)	Once between 1-2 days	NA	Fecal, saliva, vaginal	QIAamp DNA Mini Kit	Illumina HiSeq 2500	Correlation was highly significant between low *Faecalibacterium/Fusobacterium*

China	Control (n=73)<br><br>NA	before delivery		Stored immediately at -20⁰C, and subsequently storedat -80⁰C at the laboratory.	16S rRNA<br><br>V3-V4	QIIME<br><br>Greengenes	ratios and 2-hour blood glucose level.
Liu (2019) [31]<br><br>China	Cross-sectional<br><br>GDM (n=11)<br>Hyperlipidemia (n=11)<br>GDM+hyperlipidemia (n=12)<br>Control (n=11)<br><br>GDM: 29.3 (0.9)<br>Control: 28.2 (0.8)	Once between 27-33 weeks	GDM: 26.6 (1.1)<br>Control: 26.7 (0.6)	Fecal<br><br>Stored at -80⁰C	PowerMax (stool/soil) DNA isolation kit<br><br>16S rRNA<br><br>V3-V4	Illumina HiSeq4000<br><br>QIIME<br><br>SILVA128	Relative abundance of bacterial *taxa Streptococcus*, *Faecalibacterium*, *Veillonella*, *Prevotella*, *Haemophilus* and *Actinomyces* was significantly higher in diabetes plus hyperlipidemia cohorts.
Ye (2019) [14]<br><br>China	Cross-sectional<br><br>GDM (n=36)<br>Control (n=16)	Once between 24-28 weeks	GDM 1: 24.9 (3.8); GDM 2: 27.2 (3.8); Normal: 24.3 (2.9)	Fecal<br><br>Stored at -80⁰C within 2 hours of collection.	QIAamp DNA Stool Mini Kit<br><br>16S rRNA<br><br>V3-V4	Illumina MiSeq<br><br>Usearch, UCHIME, RDP classifier<br><br>Silva	*Blautia*, *Eubacterium_hallii*_group, and *Faecalibacterium* in the gut microbiota showed significant differences among the normoglycemic mother, successful glycemic control, and failure of glycemic control groups.
Gao (2020) [27]<br><br>China	Case-control<br><br>GDM (n=9)<br>Pre-pregnancy T2DM (n=8)<br>Overt diabetes (n=5)	Once at 20 weeks	Hyperglycemia: 24.3 (22.3–28.4); Normoglycemia: 20.5 (19.1–22.5)	Fecal<br><br>Stored at -80⁰C	Qiagen QIAamp DNA Stool Mini Kit<br><br>16S rRNA<br><br>V3-V4	Illumina MiSeq<br><br>UCHIME, RDP classifier<br><br>Silva (SSU123)	Women with hyperglycemia in pregnancy had an increased abundance of *Nocardiaceae*, F*usobacteriaceae*. HbA1c levels were positively correlated with *Bacteroidaceae* and *Enterobacteriaceae* and

	Normoglycemic (n=28)<br><br>Hyperglycemia: 29 (27.00–33.5); Normoglycemia:28 (25–30)						negatively correlated with *Christensenellaceae.* CRP was positively correlated with the *Bacteroidaceae* and *Fusobacteriaceae* families and the *Fusobacterium* genus.
Ma (2020) [26]<br><br>China	Case-control<br><br>GDM (n=98) Matched healthy control (n=98)<br><br>GDM:31.0 (28.8–35.0); Control: 31.5 (28.8–35)	Once between 10-15 weeks	GDM: 22.8 (3.0); Control: 20.8 (2.7)	Fecal<br><br>NA	QIAamp Fast DNA Stool Mini Kit<br><br>16S rRNA<br><br>V4	Illumina MiSeq<br><br>QIIME<br><br>Silva (SSU123)	*Eisenbergiella*, *Tyzzerella 4*, and *Lachnospiraceae NK4A136* were enriched in the GDM group, whereas *Parabacteroides*, *Megasphaera*, *Eubacterium eligens* group, remained dominant in the controls.
Mokkala (2021) [32]<br><br>Finland	Intervention<br><br>GDM (n=167) Control (n=103) 30.9 (30.5)	1st time point at average 13.9 weeks and 2nd time point at average 35.2 weeks.	29.2 (4.0)	Fecal<br><br>Stored at -20^{0}C	GTX stool extraction kit<br><br>metagenomic<br><br>NA	Illumina HiSeq<br><br>FASTQ, HUMAnN2<br><br>Silva	The composition and inferred function of gut bacteria, is not involved in the incidence of GDM in overweight and obese women.

Supplementary Table S4. Quality assessment of the prospective cohorts and cross-sectional studies (n=11)

	Ferrocino 2018 [20]	Mokkala 2017 [30]	Ye 2019 [14]	Fugmann 2015 [28]	Cortez 2018 [15]	Liu 2019 [31]	Koren 2012 [25]	Crusell 2018 [17]	Wang 2018 [16]	Gomez-Arango 2016 [29]	Kuang 2017 [18]
Q1	Y	Y	Y	Y	Y	Y	Y	Y	Y	Y	Y
Q2	Y	Y	Y	Y	Y	Y	Y	Y	Y	Y	N
Q3	NR	NR	NR	NR	NR	NR	NR	N	NR	NR	NR
Q4	Y	Y	Y	Y	Y	Y	Y	Y	Y	Y	NR
Q5	N	N	N	N	N	N	N	N	N	N	N
Q6	Y	Y	Y	Y	Y	Y	Y	Y	Y	Y	N
Q7	Y	N	N	N	N	N	Y	Y	Y	N	N
Q8	Y	N	Y	N	N	Y	Y	Y	Y	Y	N
Q9	Y	Y	Y	Y	Y	Y	Y	Y	Y	Y	N
Q10	Y	N	N	N	N	N	Y	Y	N	N	N
Q11	Y	Y	Y	Y	Y	Y	Y	Y	Y	Y	Y
Q12	NR	NR	NR	NR	NR	NR	NR	NR	NR	NR	NR
Q13	N	N/A	N/A	N/A	N/A	N/A	NR	Y	N/A	Y	N/A
Q14	Y	Y	N	N	N	N	N	Y	N	N	N
**Quality**	**Good**	**Good**	**Good**	**Fair**	**Fair**	**Good**	**Good**	**Good**	**Good**	**Good**	**Fair**

Quality of included studies was assessed using the National Institutes of Health (NIH) Quality Assessment tool for Observational Cohort and Cross-Sectional Studies [21]. Q, question; CD, cannot be determined; NA, not applicable; NR, not reported; N, no; Y, yes.

Q 1. Was the research question or objective in this paper clearly stated? **Q 2.** Was the study population clearly specified and defined? **Q 3.** Was theparticipation rate of eligible persons at least 50%? **Q 4.** Were all the subjects selected or recruited from the same or similar populations (including the same time period)? Were inclusion and exclusion criteria for being in the study prespecified and applied uniformly to all participants? **Q 5.** Was a sample size justification, power description, or variance and effect estimates provided? **Q 6.** For the analyses in this paper, were the exposure(s) of interest measured prior to the outcome(s) being measured? **Q 7**. Was the timeframe sufficient so that one could reasonably expect to see an association between exposure and outcome if it existed? **Q 8**. For exposures that can vary in amount or level, did the study examine different levels of the exposure as related to the outcome (e.g., categories of exposure, or exposure measured as continuous variable)? **Q 9.** Were the exposure measures (independent variables) clearly defined, valid, reliable, and implemented consistently across all study participants? **Q 10**. Was the exposure(s) assessed more than once over time? **Q 11**. Were the outcome measures (dependent variables) clearly defined, valid, reliable, and implemented consistently across all study participants? **Q 12.** Were the outcome assessors blinded to the exposure status of participants? **Q 13**. Was loss to follow-up after baseline 20% or less? **Q 14.** Were key potential confounding variables measured and adjusted statistically for their impact on the relationship between exposure(s) and outcome(s)?

Supplementary Table S5. Quality assessment of case-control studies (n=2)

	Ma et al, 2020 [26]	Gao et al, 2020 [27]
Q1. 1. Was the research question or objective in this paper clearly stated and appropriate?	Y	Y
Q2. Was the study population clearly specified and defined?	Y	Y
Q3. Did the authors include a sample size justification?	N	N
Q4. Were controls selected or recruited from the same or similar population that gave rise to the cases (including the same timeframe)?	Y	Y
Q5. Were the definitions, inclusion and exclusion criteria, algorithms or processes used to identify or select cases and controls valid, reliable, and implemented consistently across all study participants?	Y	Y
Q6. Were the cases clearly defined and differentiated from controls?	Y	Y
Q7. If less than 100 percent of eligible cases and/or controls were selected for the study, were the cases and/or controls randomly selected from those eligible?	NA	NA
Q8. Was there use of concurrent controls?	Y	Y
Q9. Were the investigators able to confirm that the exposure/risk occurred prior to the development of the condition or event that defined a participant as a case?	Y	N
Q10. Were the measures of exposure/risk clearly defined, valid, reliable, and implemented consistently (including the same time period) across all study participants?	Y	Y
Q11. Were the assessors of exposure/risk blinded to the case or control status of participants?	NR	NR
Q12. Were key potential confounding variables measured and adjusted statistically in the analyses? If matching was used, did the investigators account for matching during study analysis?	N	N
**Quality**	**Good**	**Good**

Q, question; CD, cannot be determined; NA, not applicable; NR, not reported; N, no; Y, yes.

Supplementary Table S6. Quality assessment of included intervention (n=1)

	Mokkala et al, 2021 [32]
Q1. 1. Was the research question or objective in this paper clearly stated and appropriate?	Y
Q2. Was the study population clearly specified and defined?	Y
Q3. Did the authors include a sample size justification?	Y
Q4. Were controls selected or recruited from the same or similar population that gave rise to the cases (including the same timeframe)?	Y
Q5. Were the definitions, inclusion and exclusion criteria, algorithms or processes used to identify or select cases and controls valid, reliable, and implemented consistently across all study participants?	Y
Q6. Were the cases clearly defined and differentiated from controls?	Y
Q7. If less than 100 percent of eligible cases and/or controls were selected for the study, were the cases and/or controls randomly selected from those eligible?	Y
Q8. Was there use of concurrent controls?	Y
Q9. Were the investigators able to confirm that the exposure/risk occurred prior to the development of the condition or event that defined a participant as a case?	Y
Q10. Were the measures of exposure/risk clearly defined, valid, reliable, and implemented consistently (including the same time period) across all study participants?	Y
Q11. Were the assessors of exposure/risk blinded to the case or control status of participants?	Y
Q12. Were key potential confounding variables measured and adjusted statistically in the analyses? If matching was used, did the investigators account for matching during study analysis?	Y
**Quality**	**Good 100**

Q, question; CD, cannot be determined; NA, not applicable; NR, not reported; N, no; Y, yes.
